# How do we sell the hygiene message? With dollars, dong or excreta?

**DOI:** 10.1186/1476-069X-9-27

**Published:** 2010-06-18

**Authors:** Peter Kjær Mackie Jensen, Pham Duc Phuc, Line Gram Knudsen West

**Affiliations:** 1 Department of International Health, Immunology and Microbiology, Faculty of Health, University of Copenhagen, Øster Farimagsgade 5 PO Box 2099.1014 Copenhagen K. Denmark; 2 National Institute of Hygiene and Epidemiology, Division of Enteric Infections, 1 Yersin Street, Hanoi, Vietnam

## Abstract

In North and Central Vietnam it is common among farmers to use excreta from the family double vault composting latrine (DVC) as fertilizer in the fields. The official Vietnamese health guidelines stipulate a six-month period of composting before applying excreta to two of their three annual crops. However, farmers in this region cannot afford to follow these guidelines and this paper presents the reasons why.

In their efforts to ensure optimal hygienic conditions, by providing a guideline, the Vietnamese health authorities have not put sufficient attention to the ‘excreta economy’ in relation to farmers’ livelihoods. The free fertilizer in the household DVC represents a value of approximately US$ 15.5 per year - or the equivalent of 15 percent of the annual household income for the poorest 20 percent of farmers. For this reason, the economic benefits derived from free fertilizer outweigh the hygiene message for most Vietnamese farmers. Even at national level the excreta economy has an impact. If Vietnam were to replace human excreta with imported fertilizer, it would involve an extra national expenditure of at least US$ 83 million a year.

In order to convince Vietnamese farmers to adopt different fertilizing methods when reusing human excreta, it is necessary for the Vietnamese health authorities to change their hygiene message. They need to replace their current health sector-specific approach with a holistic one that takes the premises of farmers' livelihoods into account. If they do not the hygiene message will simply be lost.

## Introduction

Selling the message of good hygiene and sanitation practices has been a problem for water and sanitation projects all over the world. While the appreciation of clean water is almost universal, the perception of hygiene and its connection to health and sanitation varies greatly from region to region and is often deeply rooted in local age-old customs. When receivers and senders do not share the same hygienic priorities or values, the communication of hygienic messages become very difficult.

In the northern and central rural regions of Vietnam, farmers do not follow the official Vietnamese health guidelines regarding the use of the Double Vault Composting latrine (DVC). The DVC accommodates the reuse of human excreta in the agricultural production and is the latrine type for 20.5 % of all Vietnamese households[1]. In rural areas in north and central regions nearly 100% of the households reuse excreta, either via the DVC, or the now illegal single vault latrine [2]. The farmers need cheap fertilizer and although they know that the uncomposted latrine content can be harmful to human health, they still believe that it has a very positive nutrient impact on their agricultural production[3]. Some sanitation projects in Vietnam have even failed, partly because the promoted latrines did not accommodate for use of excreta in agriculture and latrines were either forced open or broken by farmers who wanted access to the otherwise sealed off excreta[3]. The farmers' refusal to cooperate has usually been explained by the lack of local ownership in hygiene projects promoted by governmental authorities. It is our belief however that the real problem lies in the way health authorities work and think - in Vietnam and elsewhere. While specialized knowledge is essential to any sector, such knowledge needs to be supported by an understanding of the variety of factors that influence a particular population's behavioral ways. It is our contention that without a holistic understanding of farmers' livelihoods and without an interdisciplinary approach to solving hygiene and sanitation problems efforts to improve health among rural populations are largely in vain.

In three separate field studies, we have investigated different aspects of excreta reuse in Vietnam, covering a variety of topics including hygienic evaluation of the guidelines based on field trials of Ascaris eggs die-off[4], anthropological investigations of farmers perceptions of health and human excreta [5] and finally investigating agricultural practices versus health issues in the guidelines[2,6,7]. The aim of all the studies were to evaluate the hygienic problems related to the excreta reuse in Vietnam and to find the reasons why the hygiene message is so difficult to promote. We discovered that the hygiene message is currently promoted purely from a public health perspective and this is precisely why it fails. In rural societies such as the Vietnamese, cultural, agricultural, religious and not least economic factors have equal if not greater influence on people's behavior. Thus, health and hygiene arguments only have a small chance of being heard. This paper will therefore focus on the interdisciplinary approach that has to be taken in hygiene promotion, illustrated by a new area that came up in our fieldwork in Vietnam - the excreta economy - and from this perspective the paper aims to evaluate the practical use of the current guidelines for excreta reuse in the agricultural sector.

### Hygiene promotion and health

In a country like Vietnam, where rural areas are hard hit by helminth infections associated with exposure to human excreta containing worm larvae, successful hygiene promotion is vital[8]. The prevalence of helminth infections should raise particular concern, since these infections have been linked to low growth rate as well as learning difficulties among children [9,10]. Therefore these infections are important to target if the general state of health in the rural population is to be improved.

Apart from de-worming campaigns, the Vietnamese authorities attempt to combat the high levels of helminth infections by issuing guidelines that stipulate a minimum of six months' composting (retention) for the DVC content [11], this minimum period of retention time has also been recommended in other studies and reviews [12-14]. However, our studies show that only 30% of farmers compost for the required six months. The remaining 70 % state they use the excreta for at least two of their three annual crops, indicating a maximum composting period of three to four months [2]. This practice is backed by the perception held by most interviewed farmers that health issues have to be adapted to the agricultural calendar - simply because a family's livelihood depends on agood crop yield[2].

## Discussion

### Sustainability and household excreta production

Any farmer knows that when a crop is harvested, the nutrients extracted by the harvested crop need to be replaced in the soil. Phosphorus is an important nutrient. Unlike nitrogen, phosphorus cannot be drawn from the atmosphere but has to be supplied to the soil manually, either as organic manure/waste product or by adding artificial fertilizer to the soil. Farmers are obviously interested in finding cheap and sustainable ways of replacing phosphorus. In Vietnam, this is done by recycling the content of the family latrine, using it as fertilizer in the fields.

But how much phosphorus does a Vietnamese family produce, and how much artificial fertilizer do the excreta replace? A Vietnamese rice farmer living on a diet of primarily vegetarian food will typically excrete 300-500 g feces a day[13]. All excreta is mixed with ash as it is customary in Vietnam to throw one rice bowl (approximately 500 ml) of kitchen ash in the DVC after defecation to prevent smell and flies.

After the required six-month composting period, we measured a lowering of moisture in the excreta/ash - from approximately 78 % to 35 % water [4]. According to our calculations, the daily production of excreta (including ash) from one person amounted to 420 g after the composting period - or 150 kg excreta per adult person per year. This amount complies almost with the expectations of North Vietnamese farmers, who estimate a family production of 300-400 kg excreta including ash per year.

### Nutrient value of excreta in Vietnam

In our study, we found 0.7 % phosphorus in the composted excreta with ash (to be published elsewhere). With an average household size of 4.6 [15] the households would have an annual production of 500 kg composted excreta and ash, the production of phosphorus would be equivalent to 3.5 kg pure phosphorus per year. If this amount of phosphorus was to be replaced by the commercial fertilizer commonly used in Vietnam (e.g. Diamonium phosphate fertilizer with 20 % phosphorus), a family would need to buy 17.5 kg DAP fertilizer a year.

### Excreta economy - in the household

Excreta from the family latrine are not only valuable as fertilizer, as reflected in its

replacement value by a commercial fertilizer. In Vietnam, the composted excreta also has a local trading value, currently 500-700 Vietnamese Dong (VND) - or the equivalent of US$ 0.031-0.044 per kg (2008 prices). At first glance, not a high value, but for the poorest 20 % of the rural farming population, this sum accounts for as much as 15 % of the annual household income[1]. In our previous studies we saw that the DVC were evenly used between the different socio-economic groups[2].

If the price of excreta were calculated on the basis of its phosphorus value, the market price for one kg of pure phosphorus would be 71,000-100,000 VND. We need to keep in mind that this price is only the perceived value of the excreta and not based on the excreta's actual content of phosphorus. Surprisingly enough, the equivalent price for the imported Chinese DAP fertilizer is also 100,000 VND per kg phosphorus (price in Hanoi October 2008). These figures clearly reflect that the Vietnamese farmers are dealing with a commodity they know has a definite commercial value[7].

### Excreta economy - at national level

We know that 20.5 % of Vietnamese households use a DVC and most farmers reuse excreta from the latrine on their fields. We have also established the value of excreta in relation to a poor family's income. The next step is to take these calculations to a national level, calculating the value of excreta reuse to the Vietnamese economy. If the current excreta production were to be replaced with imported fertiliser, the trading value of the excreta would generate minimum:

(84 million people × 20.5% DVC users × 110 kg excreta/year (estimated as an average production for adults and children) × 0.031 US$/kg excreta) ~ **US$ 58,720,200**

If 1.9 million tons excreta were to be replaced with artificial fertilizer, it would create an additional import expenditure for Vietnam of at least US$ 83 million

## Conclusion

### Hygiene promotion - far more than a health issue

The Vietnamese health authorities want the farmers to compost their excreta for a minimum of six months inside their DVC. The farmers on the other hand need the excreta in intervals of only three-four months. To comply with regulations the farmers would have to replace the excreta with artificial fertilizer once a year. This would mean an extra expenditure for the farmer of eight % of the household income. If the authorities were to suggest a non-reuse system this figure would increase to 15 % of the annual income. It appears highly unlikely that a poor farmer in rural Vietnam would be persuaded to spend that much of his household income to promote what is to him a non-visible health benefit.

It follows therefore that the hygiene message needs to be promoted differently if it is to change fertilizing methods among Vietnamese farmers. The health authorities need to come up with new interventions that accommodate the farmers' need for inexpensive fertilizer and the public health need for composted excreta that is hygienic and non-contaminated[4].

As the case of rural Vietnam reflects, hygiene promotion is far more than a health issue. Interventions to change behavior in certain populations call for interdisciplinary approaches that take into account the complexity of the context they wish to change. This applies to other sectors that face equally complex challenges [16]. If authorities understand the cultural, social, religious and economic factors that determine a population's behavioral patterns they will be able to devise strategies that accommodate these variants. Only then will efforts to promote better health and hygiene succeed.
